# The Resilience of Final-Year Pharmacy Students and Aspects of the Course They Found to Be Resilience-Building

**DOI:** 10.3390/pharmacy10040084

**Published:** 2022-07-17

**Authors:** Lezley-Anne Hanna, Simone Clerkin, Maurice Hall, Rebecca Craig, Alan Hanna

**Affiliations:** 1 Medical Biology Center, School of Pharmacy, Queen’s University Belfast, Belfast BT9 7BL, UK; sclerkin04@qub.ac.uk (S.C.); m.hall@qub.ac.uk (M.H.); rebecca.craig@qub.ac.uk (R.C.); 2 Queen’s Management School, Queen’s University Belfast, Belfast BT9 5EE, UK; a.hanna@qub.ac.uk

**Keywords:** CD-RISC-25, pharmacy student, questionnaire, resilience

## Abstract

Background: This work aimed to investigate final year pharmacy students’ resilience (as determined by the CD-RISC-25 tool), whether students considered certain aspects of the course to be resilience-building, and the role of the university in developing this attribute. Methods: Following ethical approval and an invitation to participate, data were collected from consenting students at Queen’s University Belfast via a pre-piloted paper-based questionnaire. Descriptive statistics were performed. To ascertain significant differences (*p* < 0.05) by gender, the Welch Two Sample *t*-test was used for the CD-RISC-25 mean scores and the Mann-Whitney U Test and Chi-squared test for Section B data. Results: The response rate was 80.61% (79/98). The mean CD-RISC-25 score for males was higher (not significantly) than the female mean score (70.39 versus 67.18, *p* = 0.2355, possible score range 0–100). While 93.67% (74/79) considered the School has a responsibility to develop resilience, <20.00% availed of the free resilience building events. Activities deemed to help build resilience included being able to make mistakes in a safe environment and needing to achieve a high grade to pass assessments. Conclusions: Resilience levels among future pharmacists at Queen’s University Belfast should be improved going forward. A strategy, developed in light of conducting this research (from one institution), will now be implemented to enhance the curriculum with regard to resilience building opportunities.

## 1. Introduction

Developing resilience across the health care workforce is important and particularly relevant at the present time given the COVID-19 pandemic. Health care students typically have a high academic workload, pressure from high-stakes assessments, and exposure to diverse patient issues when out on placements. Unfortunately, students may experience mental health issues and mental distress including sleeping problems, depression, anxiety, stress, and suicide risk [1,2,3]. This can also lead to impaired cognitive functioning and burnout, which negatively impacts students’ academic performance and student experience [4,5,6]. Similarly for health care professionals, resilience can be reduced by stress, subsequently affect well-being, and potentially cause burnout. This can also negatively impact professional performance at work, including the quality of care being provided to patients and the likelihood of making errors [7,8,9]. Given the importance of this subject area, it is unsurprising that a global community of practice for pharmacy workforce resilience has recently been initiated [10].

In relation to measuring resilience, various psychometric scales have been developed. Windle and colleagues reviewed the psychometric rigor of nineteen such scales developed for use in general and clinical populations. These scales included the Connor-Davidson Resilience Scale (CD-RISC), the Resilience Scale for Adults, Brief Resilience Scale, Ego Resilience, and Psychological Resilience [11]. Overall, the CD-RISC, the Resilience Scale for Adults, and the Brief Resilience Scale received the best psychometric ratings (it should be noted that while this publication has been cited over 2000 times, it was published ten years ago) [11]. The CD-RISC is sub-divided in three versions, namely the CD-RISC-2, CD-RISC-10, and CD-RISC-25. While many research studies refer to the established scales [12], other academics continue to develop bespoke tools. For example, Chisholm-Burns and colleagues prepared a scale for use in the didactic portion of the doctor of pharmacy degree program to identify pharmacy students with a greater capacity to overcome academic adversity. The authors concluded that the final version of their Academic Pharmacy Resilience Scale (APRS-16) was a reliable and valid tool for measuring academic resilience in pharmacy students [13].

In terms of published work in a pharmacy context, students attending three schools of pharmacy in the United Kingdom (1161 respondents) completed psychometric measures of academic resilience and well-being. The authors found that academic resilience was higher in first-year students than in other years and that it varied by pharmacy school and gender but not ethnicity [14]. Other studies have focused on ways to build resilience. In a pharmacy-specific context, one study conducted in the United States of America (USA) explored ways to build resilience among pharmacists [15]. In Australia, Monash University embedded the development of resilience skills into its undergraduate programs [10]. In New Zealand, the University of Otago recently introduced a workshop into the fourth year of the undergraduate pharmacy curriculum. [16]. Other educators have endeavored to improve and restore the well-being and resilience of pharmacy students during the COVID-19 pandemic [17].

Conducting a study to ascertain the level of resilience among Queen’s University Belfast final-year pharmacy students should strengthen the existing evidence base, particularly from a UK context. Other than the few studies outlined above, most research about resilience in health care students relates to medical or nursing students. The findings of this work have helped inform the development of school strategies and approaches to support future pharmacists to improve their resilience and well-being, particularly as they prepare to join the workforce.

The aim was to investigate Queen’s University Belfast Year 4 MPharm students’ level of resilience (as determined from the CD-RISC-25) and ascertain their views on the role of the School of Pharmacy in developing this attribute. The study also sought to investigate whether students considered certain aspects of the MPharm course to be resilience-building and if gender affected responses.

## 2. Materials and Methods

The subjects were Year 4 students enrolled in the Queen’s University Belfast MPharm degree programme in 2021–2022 (n = 98, excluding the research student S.C.). This year group was chosen at it was soon to sit their final examinations, graduate, and enter the workplace.

Data were collected by means of a self-completed paper-based questionnaire. Students were informed that they could change their mind and withdraw from the study, without giving a reason, up to the point when they handed in their questionnaire. The participant information sheet and questionnaire cover sheet included a statement explaining that there would be no consequences if they chose not to participate or any advantages or rewards for completing it. The questionnaire cover sheet asked students to indicate that they gave their consent to take part in the research study.

The questionnaire was developed with reference to other published work in the area [10,12,14,18] and consists of three sections. Section A was the CD-RISC-25 (n = 25 items) and consists of statements describing different aspects of resilience. As described by the authors of the CD-RISC-25 [12], it encompasses items that primarily measure a particular parameter, although there can be some overlap. The parameters are “hardiness, coping, adaptability/flexibility, meaningfulness/purpose, optimism, regulation of emotion, and cognition and self-efficacy” [12]. Section B (17 items) primarily related to the role of Queen’s University Belfast School of Pharmacy in developing resilience among pharmacy students and whether various elements of the MPharm were deemed helpful in building resilience. These statements were informed by the authors’ previous work about stress and stressors among MPharm students [18]. Section C had one question about gender (i.e., no identifiable information was requested in the questionnaire). Age and ethnicity were not sought as these parameters have the potential to uniquely identify students. To maximize response rates, the questions were mainly closed-ended with participants asked to select an option from a 5-point rating scale [19].

With regard to piloting, firstly, it should be noted that Section A (CD-RISC-25) has been used in numerous studies across the globe, and the authors of the CD-RISC-25 clearly stipulate that the scale’s content may not be modified, i.e., question wording and order must not be altered, and it is not permissible to add or remove any of the 25 items or alter the scoring choices [12]. There were also restrictions in relation to sharing it. The entire questionnaire was piloted on five Queen’s University Belfast colleagues. In addition, the questionnaire without Section A (CD-RISC-25) was piloted on five PhD students and post-doctoral staff, with a brief description of Section A provided for context. As a result of the pilot, minor amendments were made to Section B.

The distribution of the paper-based questionnaire occurred in November 2021 in a compulsory class. Students were sent an email invitation and participant information sheet in advance (but not the questionnaire). Questionnaire distribution only occurred once (i.e., there were no follow-up visits).

In terms of data analysis, coded responses from the completed questionnaires were entered into Microsoft Excel^®^ version Office 365 (Microsoft Corporation, Redmond, WA, USA) in January 2022. The CD-RISC-25 authors’ instructions on scoring were adhered to [12]. The scoring of CD-RISC-25 is based on summing the total of all items, each of which is scored from 0 to 4, with the possible scoring range therefore being 0 to 100. Higher overall scores reflect greater resilience. Otherwise, questionnaire analysis mainly took the form of descriptive statistics, such as frequencies and percentages. Section B data were largely non-parametric in nature (nominal or ordinal data), and therefore, for any inferential statistical analysis (comparisons of responses by gender with *p* < 0.05), appropriate statistical tests such as the Chi-squared and Mann–Whitney U-test were employed. The open-response question (in Section B) was analyzed using thematic analysis [20].

## 3. Results

### 3.1. Response Rate and Demographic Information (Section C of the Questionnaire)

The response rate for the questionnaire was 80.61% (79/98). Out of the 79 respondents, 78 completed the questionnaire in its entirety, and 1 omitted a few statements. The number of respondents who completed each statement is specified throughout. If readers wish to peruse the raw data from the 79 individual questionnaires, these are provided within a spreadsheet in Supplementary Material S1. For the one demographic information question, the four verbatim options that respondents could choose from were: Male, Female, Prefer Not to Say, and Other (please fill in the blank). Out of the 79 respondents, 23 reported being male (29.11%), 55 reported being female (69.62%), and 1 omitted to select any of the options (1.27%).

### 3.2. CD-RISC-25 (Section A of the Questionnaire)

To avoid any violation of copyright, the CD-RISC-25 items are not included in this paper. As previously mentioned, more information about the CD-RISC-25 tool can be obtained directly from its authors [12]. The students’ mean score, standard deviation, and median scores for the CD-RISC-25 are provided in Table 1. As can be seen, males had a higher overall mean score than females. The Welch two-sample *t*-test was carried out to compare the male and female overall mean scores, but there was no significant difference between them (*p* = 0.2355, *t* = 1.2036, df = 41.904).

### 3.3. The Role of the QUB School of Pharmacy in Building Resilience and Whether Certain Aspects Helped Build Resilience (Section B of the Questionnaire)

The findings from the first question (12 items) in Section B are shown in Table 2 below, i.e., the twelve verbatim statements from the questionnaire and their corresponding responses. Please note, after the Mann–Whitney U test, there were no significant differences in male and female responses for any of these statements. The respondents deemed resilience to be an important characteristic for a future pharmacist to have and that the QUB School of Pharmacy has a responsibility to develop it. The statements with the highest interpolated median scores (>4) for resilience growth were: having to achieve a high grade to pass, having to pass gateway assessments such as objective structured clinical examinations (OSCEs), being given opportunities within the degree to make mistakes in a simulated environment and learn from them, and having to complete work-based placements. The lowest-scoring statement related to the support and advice they had received from their personal tutor.

The questionnaire then asked respondents to rate their resilience at two different time points. The scales stated that 1 equaled low resilience and 10 equaled high resilience. The verbatim question from the questionnaire was: This question asks you to think about your level of resilience at different time points in the degree program: (a) On the scale of 1 to 10 provided below, rate your level of resilience at the start of the MPharm degree (tick the relevant box); (b) On the scale of 1 to 10 provided below, rate your level of resilience now (tick the relevant box).

**Figure 1:**
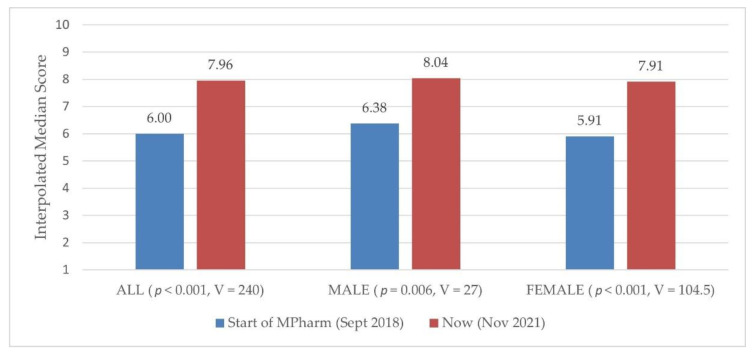
Students’ self-reported resilience levels (interpolated median scores) at the start of the MPharm degree and ‘now’, with *p* and Wilcoxon signed-rank V-statistic values included (n = 78). The scale was 1 to 10, where 1 equaled low resilience and 10 equaled high resilience.

Notably, the start of the MPharm degree for the majority of respondents was September 2018. A minority started in September 2017 but then had to repeat a year at some point during the course. ‘Now’ represented the time of questionnaire completion, which was November 2021 (their final examinations are in May 2022)]. The interpolated median scores and p values are provided in Figure 1. As can be seen, the respondents had a higher score ‘now’ than at the start of the MPharm degree. Males had a higher score than females at the start of the MPharm degree (6.38 versus 5.91) and ‘now’ (8.04 versus 7.91). A bigger numerical difference between the two time points was seen for females (5.91 at the start and 7.91 now = 2 compared with a difference of 1.66 for males).

**Figure 2:**
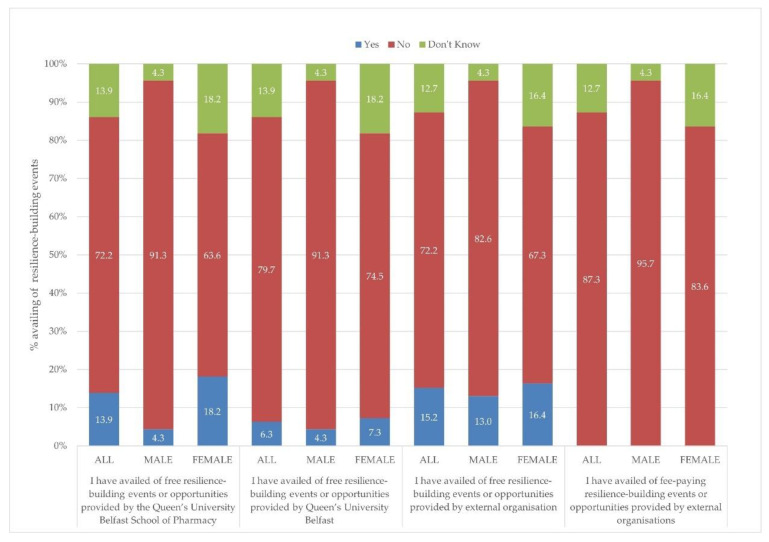
Students’ responses (n = 78) about whether they had availed themselves of resilience-building events or opportunities offered by QUB, QUB School of Pharmacy, and external organizations (free and fee-paying).

The third question in this section asked respondents about their participation in resilience-building events. The verbatim statements from the questionnaire and the responses are illustrated in Figure 2. The main finding, which is evident from Figure 2, is that the majority of student respondents had not availed themselves of any resilience-building events. Females were more likely to report that they had availed themselves of free resilience-building events or opportunities provided by the QUB School of Pharmacy than males (18.2% females selected ‘yes’ compared with 4.3% males; *p* = 0.047, df-2, X^2^ = 6.131).

Finally, Question 4 in Section B gave the students an opportunity to provide any other comments they wanted on resilience. A summary of these open responses is provided below (see Table 3) and all respondents who provided responses to this question are included. The verbatim statement from the questionnaire was: Is there is anything else you want to add about resilience? If so, please use the space below.

## 4. Discussion

In this study, the mean resilience score (obtained via the CD-RISC-25) for the respondents was 68.01. This was higher than that of Irish university students (n = 83) at Trinity College Dublin measured during a four-month long randomised control trial in 2019 [21] and three other studies involving Chinese medical students [22,23,24] with sample sizes of 2069, 1722 and 1266. It was lower than that reported for senior-level baccalaureate nursing students (n = 27) in North Carolina [25]. Male respondents in this study had a higher overall CD-RISC-25 score than females, reflecting similar results from Chinese medical students [22], Canadian medical students [26] and Nigerian nursing students [27]. The score obtained in this study is similar to a mean score obtained over ten years ago by psychiatric outpatients in the USA (i.e., 68.0) [12]. However, it should be noted that at the time of conducting the research, the student respondents were still exposed to stressors linked to the COVID-19 pandemic. Additionally, Cassidy and colleagues found that academic resilience and wellbeing were significantly lower in UK pharmacy students compared with other student populations (although not via CD-RISC-25) [14].

All students in this current study were in agreement that resilience is an important characteristic to have as a future pharmacist and that QUB School of Pharmacy has a responsibility to help develop this. The role of resilience in higher education has been explored in another study involving nursing students [28]. Unfortunately, the results showed that fewer than 30% of the student respondents availed themselves of extracurricular resilience-building events during their MPharm degree, suggesting they did not know about them, could not attend at the specified time and/or did not consider them worth attending. It is also interesting to note that even though female participation at the school or university-led resilience building events was higher than males, females still had a lower CD-RISC-25 mean score than male students. A Cochrane Review evaluated psychological interventions to foster resilience in health care students. Interventions included mindfulness, coaching, active coping by problem-solving, cognitive behavioral therapy and positive psychology. Compared with controls, the authors found very low-certainty evidence that health care students receiving resilience training may report higher levels of resilience (9 studies, 561 participants), lower levels of anxiety (7 studies, 362 participants) and lower levels of stress or stress perception (7 studies, 420 participants). The authors conclude that study designs need to be improved before firm conclusions can be reached [29]. Similarly, Seo and colleagues conducted a systematic review to ascertain the efficacy of resilience curricula in undergraduate and postgraduate medical education. Eight of the twenty-one studies involved undergraduate students (n = 598 students), and thirteen involved postgraduate students (n = 778 students). They reported extensive discrepancies in the duration, delivery and curricular topics and concluded that more research is needed to build optimal methods that foster resilience [30].

In addition to the individual studies [15,16,17] and systematic reviews [29,30], supporting medical students to build coping strategies and reserves has been shown to enhance professionalism and the quality of patient care in addition to resilience [31]. Baumgartner and Schneider found that mindfulness activities appeared to improve the grades of participating students as well as promoting the development of resilience [32]. For extracurricular events, explicit linkage to resilience may increase engagement. Greater awareness could also be achieved if event-promoting posters were accessible in student bars and the Students’ Union, coupled with eye-catching banners on university online platforms and social media sites [33]. There are other things students can do in their own time as part of self-care by taking part in resilience-building activities such as reflective journaling, mindfulness walks, meditation, aromatherapy, and muscle relaxation [34].

The self-reported resilience levels of respondents increased from the start of the MPharm degree (September 2018) up to the point when the questionnaire was completed (November 2021), which is encouraging. The statements with the highest interpolated median scores (>4), and hence deemed to help build resilience, were: having to achieve a high grade to pass, having to pass gateway assessments such as OSCEs, being given opportunities to make mistakes in a simulated environment and learn from them, and completing work-based placements. The lowest scoring statement related to the support and advice they had received from their personal tutors. Some common factors about these professional classes and OSCEs are that students work independently in a time-bound environment. It is very easy to score zero if the student does something that would compromise patient safety (e.g., a dosing calculation error or failing to spot a drug–drug interaction of the prescription that could result in serious harm). When students obtain zero or a low score, they are encouraged to reflect and complete an electronic error log. Unsurprisingly, student respondents valued the chance to undertake formative assessments (dispensing and checking prescriptions in a simulated mock pharmacy) and receive feedback prior to starting the summative assessments. It should be noted that experiential learning placements in practice will increase in amount across all UK MPharm degrees following the publication of the latest General Pharmaceutical Council (GPhC) Standards for the Initial Education and Training of Pharmacists [35].

It is important that MPharm course providers ensure that their future pharmacists are being trained to become safe and effective health care practitioners who provide high-quality person-centred care. This means that future pharmacists need to have an ability to bounce back from difficult patient interactions and be able to quickly shift their focus and attention to other patients. Yet we are witnessing pharmacy students who appear to not be able to do this in an academic context. For example, minor delays to the start of an assessment (that had no impact on assessment duration) have caused some students to report feeling excessive levels of stress that negatively affected their concentration and academic performance. Other authors have started to investigate new measures of academic tenacity in terms of student success and assert that this will be as important (if not more so) as other measures such as grit [36]. It would be helpful if the professional pharmacy organization in Northern Ireland considered ways to support pharmacists to manage stress and grow resilience, particularly with interventions targeted at early career pharmacists. The new GPhC Standards for the Initial Education and Training of Pharmacists [35] give pharmacists independent prescribing rights. The level of responsibility that future pharmacists will have as autonomous prescribers managing patients with diverse and complex needs, and at such an early stage in their career, will be much greater than at present.

In terms of strengths and limitations, this study’s strengths were that it used a validated resilience-specific measuring tool (CD-RISC-25), achieved a high response rate (80.6%) and involved pharmacy students, who are underrepresented in this field in comparison with medical and nursing students. With permission obtained to use the CD-RISC-25 [12] and adaptations to the statements in Section B to suit a particular context, this questionnaire-based study could be transferred to other education settings or other year groups. The study had some significant limitations in that it was from a single year group in a single site and so the findings cannot be generalized to broader populations, and the authors acknowledge they are may not be representative of pharmacy students enrolled elsewhere. Moreover, the authors did not receive any funding to conduct this work, and therefore we prioritized use of the CD-RISC-25 to be among final-year students. Furthermore, opinions were only captured at one point in time. The CD-RISC-25 mean score may have varied if the study had been carried out closer to the final-year written exams (although we would not have wanted to distract students from their revisions to invite them to participate in a study) or during their semester break (which probably would have yielded a poor response rate). Readers are encouraged to take these weaknesses into account, and this is why we have opted to include it as a ‘Communication’ rather than an ‘Article’.

### Ten Approaches That QUB School of Pharmacy Intend to Implement in Light of Undertaking This Work

1. To help students develop skills, we will set expectations and context for the year ahead through a formal induction. We will aim to provide greater scaffolding and support in the earlier years but taper this back as the students progress into third and final year.
2. While we have a role to play in building students’ resilience, there is still a lack of evidence outlining the best way to do this. Any training sessions or multicomponent interventions about resilience that we add into the course should be evaluated to determine their short- and longer-term success.
3. Encourage and facilitate students to reflect, including suggesting that they may want to keep a journal.
4. Introduce productive meetings for students with their personal tutors about ways to build resilience and confidence. Help personal tutors to appreciate where support is available for students’ mental health and well-being, and help them with professional and personal development.
5. Develop guidance about assessments (including how to prepare, perfectionism, and dealing with stress, pressure, failure and setbacks) and produce an assessment and feedback calendar for students.
6. Increase the amount of peer support and encouragement available through the formation of communities of practice and networks.
7. Expand the School of Pharmacy Mental Health and Well-Being team’s scope to include resilience ambassadors (alongside the student mental health first-aiders).
8. Review the learning and teaching strategy to ensure that our approaches enable students to become competent and confident through active learning opportunities. Investigate the balance between instructor-led teaching and peer- or self-directed learning and discovery. Review the interprofessional learning material to ensure that it encompasses real-world issues.
9. Review the assessment strategy to ensure there is adequate formative assessment to allow students to make mistakes in a safe environment and learn from them. Consider the justification for using a range of pass marks and think more holistically about the assessment burden
10. Ensure that resilience-building events have explicit links to resilience and are promoted adequately. Recommend relevant resources, such as the podcast series ‘The Resilient Pharmacist’ [37].

## 5. Conclusions

The authors deem that resilience levels among future pharmacists at Queen’s University Belfast need to be improved going forward. While it is hoped that resilience will increase generally post-pandemic, a strategy (developed in light of conducting this research at one institution and outlined in the Discussion section of this paper) will now be implemented to enhance the curriculum and students’ ability to develop resilience.

## Figures and Tables

**Table 1:** CD-RISC-25 mean score, standard deviation, and median score for the 79 respondents, with subdivisions showing male and female scores. Possible score range was 0–100.

	All	Male	Female
Mean score	68.01	70.39	67.18
Standard deviation	10.82	10.69	10.86
Median score	67	71	66

**Table 2:** Views of the respondents (n = 79) on the role of the School of Pharmacy in building resilience and resilience-building elements.

Verbatim Questionnaire Statements from Section B, and in the Exact Order They Were Asked	SA * n (%)	A * n (%)	NAD * n (%)	D * n (%)	SD * n (%)	Interpolated Median
a. Resilience is an important characteristic for me to have as a future pharmacist	49 (62.03)	30 (37.97)	0 (0.00)	0 (0.00)	0 (0.00)	4.69
b. Queen’s University Belfast School of Pharmacy has a responsibility to develop resilience among future pharmacists	36 (45.57)	38 (48.10)	3 (3.80)	1 (1.27)	1 (1.27)	4.41
c. My level of resilience has grown because elements of the MPharm degree have required me to attain a high standard to pass (e.g., Proprietary Dispensing)	33 (41.77)	34 (43.04)	7 (8.86)	5 (6.33)	0 (0.00)	4.31
d. My level of resilience has grown because the MPharm degree has required me to successfully complete gateway assessments (e.g., progression OSCEs **)	25 (31.65)	38 (48.10)	14 (17.72)	2 (2.53)	0 (0.00)	4.12
e. My level of resilience has grown because the MPharm degree has required me to complete placements in the workplace (e.g., in community and hospital practice)	22 (27.85)	36 (45.57)	14 (17.72)	5 (6.33)	2 (2.53)	4.01
f. My level of resilience has grown because the MPharm degree has required me to undertake team tasks, including in an interprofessional context	17 (21.52)	40 (50.63)	17 (21.52)	3 (3.80)	2 (2.5)	3.94
g. My level of resilience has grown because I have had opportunities within the MPharm degree to make mistakes in a simulated environment and learn from them ***	29 (37.18)	37 (47.44)	10 (12.82)	2 (2.56	0 (0.00)	4.23
h. My level of resilience has grown because I have had to learn remotely on my own (e.g., via recorded lectures)	22 (27.85)	33 (41.77)	12 (15.20)	10 (12.66)	2 (2.53)	3.97
i. The constructive feedback I have received from academic staff has enhanced my level of resilience ***	18 (23.08)	38 (48.72)	13 (16.67)	6 (7.70)	3 (3.85)	3.95
j. The support and advice I have received from my personal tutor has enhanced my level of resilience	9 (11.39)	22 (27.85)	23 (29.11)	9 (11.39)	16 (20.25)	3.13
k. Stressors outside the MPharm degree have made me more resilient than those within the MPharm degree	23 (29.11)	24 (30.38)	23 (29.11)	7 (8.86)	2 (2.53)	3.81
l. I bounce back from setbacks I am faced with during the MPharm degree (i.e., MPharm-related setbacks)	19 (24.05)	46 (58.23)	10 (12.66)	4 (5.06)	0 (0.00)	4.05

* SA = strongly agree, A = agree, NAD = neither agree nor disagree; D = disagree and SD = strongly disagree; ** OSCE stands for objective structured clinical examination; *** 1 missing response (i.e., 78 not 79).

**Table 3:** Open response comments about resilience.

Theme	Respondent Quote (R = Respondent Number)
Goal setting and reflection	“*One of the ways of establishing resilience was by establishing goals and keeping a diary for reflection.*” (R66)
Having to take personal responsibility for developing resilience	“*Not 100% sure the university prepares us for the stress/workload associated with being pharmacists. Speaking to pharmacists in work they say they’ve had to learn this themselves…*” (R27)
Having a safe environment to learn/ the importance of formative assessments)	“*I feel I would like to build resilience in an environment that isn’t assessed. This just adds pressure and I beat myself up afterwards, but if unassessed I commend myself on going and taking part.*” (R3)
Realistic link to practice within teaching	“*More IPL* [interprofessional learning] *realistic scenarios with other students would help build resilience.*” (R14)
Health and well-being and the impact on resilience	“*A health condition has reduced my level of resilience at times, both within and outside the MPharm degree. However, I still feel I am a hard worker and determined to keep going.*” (R49) “*COVID pandemic has definitely increased the need for the development of my resilience.*”(R62)
Personal issues	“*Issues at home and the amount of pressure I put on myself* etc. *provided me with more resilience than anything related to university.*” (R25)
Other resilience-building aspects of the MPharm degree program	“*I think extemporaneous dispensing in Level 2 of the MPharm degree really helped me to build resilience.*” (R54) “*Building resilience in MPharm started in Level 2 with extemporaneous dispensing.*” (R69)
What resilience means	“*In terms of resilience in pharmacy students, I feel like it is mostly about being able to balance everything you have on in uni, as well as other aspects of your life.*” (R48)

## Data Availability

Data are contained within the article or the Supplementary Material. The data presented in this study are available in Spreadsheet S1 (i.e., the data from the individual questionnaires is within a spreadsheet within the Supplementary Material).

## Supplementary Materials

The following are available online at https://www.mdpi.com/article/10.3390/pharmacy10040084/s1, Spreadsheet S1: Questionnaire data.
